# Cell Type-Specific Annotation and Fine Mapping of Variants Associated With Brain Disorders

**DOI:** 10.3389/fgene.2020.575928

**Published:** 2020-12-03

**Authors:** Abolfazl Doostparast Torshizi, Iuliana Ionita-Laza, Kai Wang

**Affiliations:** ^1^Raymond G. Perelman Center for Cellular and Molecular Therapeutics, Children’s Hospital of Philadelphia, Philadelphia, PA, United States; ^2^Department of Biostatistics, Columbia University, New York, NY, United States; ^3^Department of Pathology and Laboratory Medicine, Perelman School of Medicine, University of Pennsylvania, Philadelphia, PA, United States

**Keywords:** genome-wide association study, schizophreina, fine mapping, variant annotation, brain disorders

## Abstract

Common genetic variants confer susceptibility to a large number of complex brain disorders. Given that such variants predominantly localize in non-coding regions of the human genome, there is a significant challenge to predict and characterize their functional consequences. More importantly, most available computational methods, generally defined as context-free methods, output prediction scores regarding the functionality of genetic variants irrespective of the context, i.e., the tissue or cell-type affected by a disease, limiting the ability to predict the functional consequences of common variants on brain disorders. In this study, we introduce a comparative multi-step pipeline to investigate the relative effectiveness of context-specific and context-free approaches to prioritize disease causal variants. As an experimental case, we focused on schizophrenia (SCZ), a debilitating neuropsychiatric disease for which a large number of susceptibility variants is identified from genome-wide association studies. We tested over two dozen available methods and examined potential associations between the cell/tissue-specific mapping scores and open chromatin accessibility, and provided a prioritized map of SCZ risk loci for *in vitro* or *in-vivo* functional analysis. We found extensive differences between context-free and tissue-specific approaches and showed how they may play complementary roles. As a proof of concept, we found a few sets of genes, through a consensus mapping of both categories, including *FURIN* to be among the top hits. We showed that the genetic variants in this gene and related genes collectively dysregulate gene expression patterns in stem cell-derived neurons and characterize SCZ phenotypic manifestations, while genes which were not shared among highly prioritized candidates in both approaches did not demonstrate such characteristics. In conclusion, by combining context-free and tissue-specific predictions, our pipeline enables prioritization of the most likely disease-causal common variants in complex brain disorders.

## Introduction

With the advent of technologies such as SNP genotyping arrays or next-generation sequencing in genome-wide association studies (Levinson et al., 2012), common variants can be reliably identified and have been associated with a large number of complex diseases (Buniello et al., 2019). However, GWAS usually detect proxy markers that are associated with diseases or phenotypic traits, and the causal functional variants may differ from the proxy markers found in GWAS (Visscher et al., 2017; Tam et al., 2019). Genotype-phenotype relationships gleaned from resources such as large-scale population genetics studies including the Genotype-Tissue Expression (GTEx) project (Consortium, 2013), empirical observations to understand genetic mechanisms underlying gene expression (Gamazon et al., 2015), and statistical models (Edwards et al., 2013; Li et al., 2017) to predict functional consequences of genetic variations have provided valuable knowledge to pinpoint putative disease causal mutations. The majority of complex diseases are context-specific in that not every tissue is equally vulnerable to the genetic variation, while most of the available predictive measures do not take into account such information. For example, in the case of neuropsychiatric diseases such as schizophrenia (SCZ), genetic variants with transcriptional effects in the central nervous system may have small or no effects in other tissues, so tissue-specific information can facilitate the identification of variants that play causal roles in disease pathogenesis (Skene et al., 2018; Mendizabal et al., 2019; Doostparast Torshizi et al., 2020). Moreover, genetic variations are known to play a central role in conferring susceptibilityof autism spectrum disorders (ASDs) and other neurodevelopmental disabilities (Sanders et al., 2015; Doostparast Torshizi et al., 2018; Grove et al., 2019). Prior studies on ASDs implicate enrichment of disease risk genes in excitatory glutamatergic neurons in the cortex as well as certain neurons in the striatum (Parikshak et al., 2013; Chang et al., 2015). Notably, a recent study (Satterstrom et al., 2020) indicates that within the heterogeneous population of cells in the human cortex, early excitatory neurons express most of the ASD risk genes, whereas microglia and choroid plexus express the fewest number of genes among the constituent cell-types. Of note, oligodendrocyte progenitor cells and astrocytes were found as the only non-neuronal cell-types to enrich for ASD risk genes. Beyond neuropsychiatric and neurodevelopmental disorders, other major debilitating brain diseases such as Alzheimer’s disease (AD) are found to be highly cell-specific. Mild memory loss is the onset of ASD culminating in severe cognitive impairments (Hardy and Selkoe, 2002; Masters et al., 2015). A recent study (Mathys et al., 2019) on transcriptional patterns of AD patients has revealed excessive enrichment of differentially expressed genes in excitatory and inhibitory neurons while demonstrating meager enrichment in microglia in prefrontal cortex. In addition, common genetic variants in AD are found to enrich in genes involved in endocytic pathways (Lambert et al., 2013; Huang et al., 2017; Grubman et al., 2019). Collectively, these observations illustrate how genetic variants predispose specific cell-types in the human brain to the disease risk which makes it necessary to further focus on context-specific measures to analyze genetic variants as opposed to conventional context-free frameworks. Convergence of context-free vs. tissue-specific fine mapping of genetic variations is a challenging task, which requires comprehensive evaluation of state-of-the-art methods to illustrate the differences and similarities in ranking mutations regarding their functional consequences.

Availability of tremendous amount of genomic data mandates creating computational pipelines to reliably extract useful knowledge with the goal of understanding how genetic mutations impact human health and phenotypic traits. Although many predictive approaches have been proposed over the past few years, it is still essential to create a standardized framework to assess and compare how context-specific fine mapping methods compare to more traditional context-free measures. In this paper, we introduce an evaluation pipeline to study the performance of general and tissue/cell-specific methods to measure the functional consequences of common genetic variants. Given the rich resources of available common variants on SCZ, we will leverage these resources to demonstrate the utility of the proposed pipeline tomake more confident prioritization of genetic variants for further downstream analyses.

## Results

### Analytical Framework on Using Context-Free and Context-Specific Methods to Find Causal Variants

The genetic basis of SCZ has been investigated extensively in recent years, leading to a large collection of common genetic variations that explain a significant fraction of the disease heritability (Purcell et al., 2014; Skene et al., 2018). Although these associations can be informative, it has proven difficult to identify “actionable” genes (Doostparast Torshizi et al., 2019) as they tend to reside in noncoding regions and act as proxy variants in linkage disequilibrium (LD) with the true causal variants. Therefore, mapping the actual disease-associated common variations to the disrupted cell-type or tissue in the context of the underlying disease, i.e., fine mapping, is crucial to prioritize causal mutations and causal genes that are relevant to the disease pathogenesis. Such an investigation guides subsequent experimentations for functional assessments and therapeutic development based on the causal genes and pathways.

**FIGURE 1:**
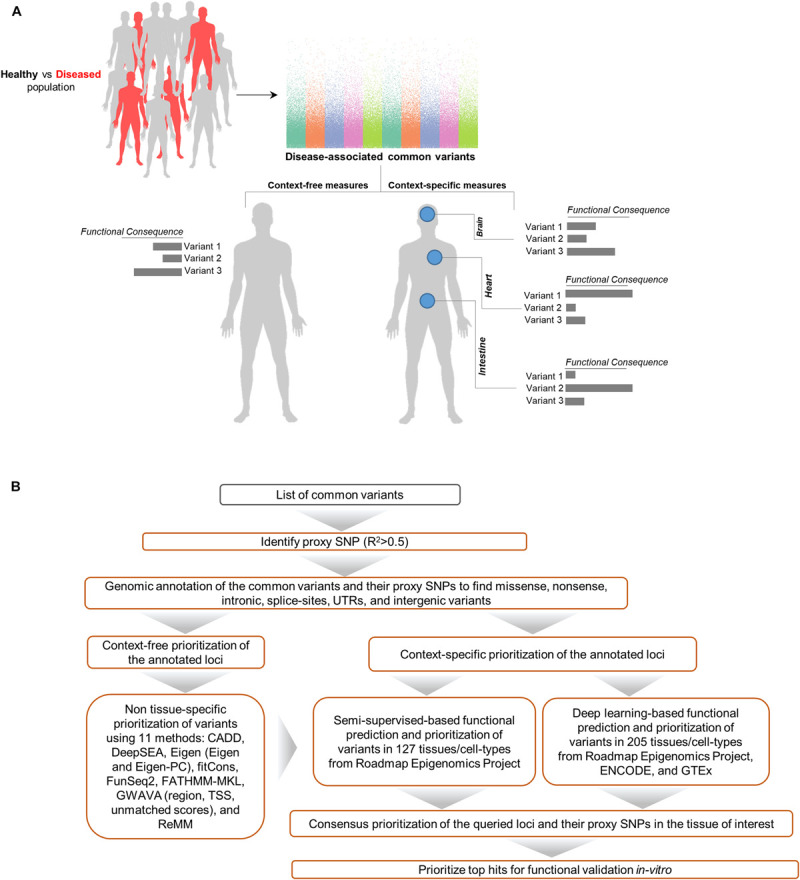
Schematic of methods for characterizing functional consequences of common genetic variants. **(A)** Overview of context-free vs. context-specific measures. **(B)** The overall structure of the present study on SCZ GWAS loci and their proxy SNPs.

Aimed at creating a general framework to evaluate and compare computational approaches for predicting functional consequences of common variants, we designed a multi-stage pipeline (Figure 1) for fine mapping of common variants across tens of cell types and tissues, leveraging a wide range of context-free and tissue-specific methods for functional annotation of GWAS loci. Although we focus on SCZ as the disease model for investigation in the study, this framework is general and can be effectively utilized in other diseases as well. The proposed pipeline starts with identifying all proxy SNPs for each of the queried common variants which are in linkage disequilibrium (LD) in the European ancestry (by default). We used correlation threshold of *R*^2^ > 0.5 by default, however users may change the threshold as desired and change to a different population group (based on Phase 3 of the 1000 Genomes Project). In the next step, we apply a filtering stage during annotation of the loci. Using annotation tools such as ANNOVAR (Wang et al., 2010), we classify variants into separate subgroups based on their predicted functional consequences such as missense, nonsense, intronic, splice-site, UTRs, exonic, and intergenic variants. Upon narrowing down the list of candidate loci, we take three separate strategies in parallel to identify disparities between context-free and cell/tissue-specific methods including: (i) context-free prioritization of the annotated loci using 11 different methods; (ii) context-specific prioritization of the annotated loci which encompasses two machine learning algorithms including (a) functional prediction and prioritization of SCZ variants in 127 tissues/cell-types from Roadmap Epigenomics Project (Bernstein et al., 2010) using a semi-supervised machine learning algorithm (He et al., 2018), (b) deep learning-based functional prediction of the variants in 205 tissues/cell-types from Roadmap Epigenomics Project (Bernstein et al., 2010), encyclopedia of DNA elements (ENCODE) (Consortium, 2012), and genotype-tissue expression, GTEx project (Consortium, 2013); (iii) consensus prioritization of the variants in the tissue of interest to prioritize the queried variants or their potential disease causing proxies.

Traditional context-free measures rank genetic loci without taking into account what tissues or organs the disease affects. To gain a better insight into the (dis) similarities of such approaches, we have used 11 distinct methods. Additionally, we have used two machine learning algorithms which estimate functional consequences of genetic loci in tens of different tissues and cell types. The methods used in this study for benchmarking are among the widely used measures in the scientific community. These methods cover conventional context-free measures as well as state-of-the-art machine learning-based context-specific methods to predict the functional consequences of genetic variants. Next, we will rank the analyzed variants through a maximum consensus procedure and create an overall ranking for each locus. Upon completion of this stage, we will have a repertoire of rankings for each locus which will then be used for comparison. Upon obtaining a final ranking of the loci, we conduct a position-specific analysis of the queried variants and their proxies to gain further knowledge which may not have been captured by any of the methods. In 2018, Pardinas et al. (2018) reported a new genome-wide association study of schizophrenia (CLOZUK GWAS; 11,260 cases and 24,542 controls), and through meta-analysis with independent PGC datasets they identified 50 novel associated loci and 145 loci in total associated with schizophrenia. In the present study, we used SCZ GWAS data from the CLOZUK. We also included the data from PGC study, though it has a significant overlap with the CLOZUK data. We acknowledge that the same approach may be applied to PGC2 study on SCZ, once the results become publicly available.

### Cell-Specific Fine Mapping of SCZ GWAS Loci

We obtained the SCZ GWAS data from CLOZUK (Pardinas et al., 2018) and PGC (Schizophrenia Working Group of the Psychiatric Genomics Consortium, 2014) studies, currently the largest SCZ GWAS consortiums. We annotated these variants using ANNOVAR (Wang et al., 2010) to characterize the exact position of each SNP. Among these variants, 67 SNPs were intergenic and 63 were intronic while the rest were UTR5′, UTR3′ and ncRNA-intronic. Only two SNPs were exonic. For cell-specific *in-silico* fine mapping of the SCZ GWAS loci, we utilized two state-of-the-art methods which predict the functional consequences of genetic variants across a wide range of cell-types and organ tissues including GenoNet (He et al., 2018) and ExPecto (Zhou et al., 2018). Since these SNPs are likely not the causal variants, but in LD with the causal variants, we also annotated the proxy SNPs, identified using LDproxy (Machiela and Chanock, 2015). In total, we identified 1,258 proxy loci (Supplementary Table S1).

**FIGURE 2:**
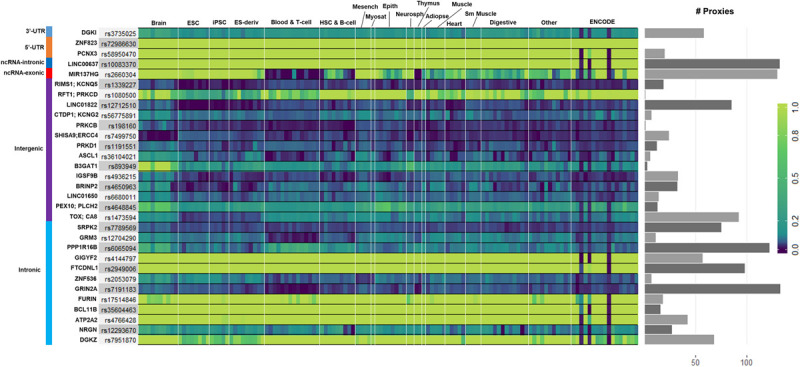
Distribution of the highest GenoNet scores on GWAS loci in which 20 loci bear the highest score uniquely in the brain and 11 loci share the highest score between the brain tissues and other tissues or cell-types. For each SNP, the corresponding gene and the genomic position of each mutation is provided. Gray bars represent the proxy SNPs of the queried variants. ESC, embryonic stem cell; iPSC, induced pluripotent stem cell; ES-deriv, embryonic stem cell-derived cultured cells; HSC, hematopoietic stem cells; Mesench, mesenchymal stem cells; Myosat, muscle satellite cultured cells; Epith, epithelial cells; Neurosph, brain-derived primary cultured neurospheres; Adiopse, adiopse nuclei; Sm Muscle, smooth muscle.

**FIGURE 3:**
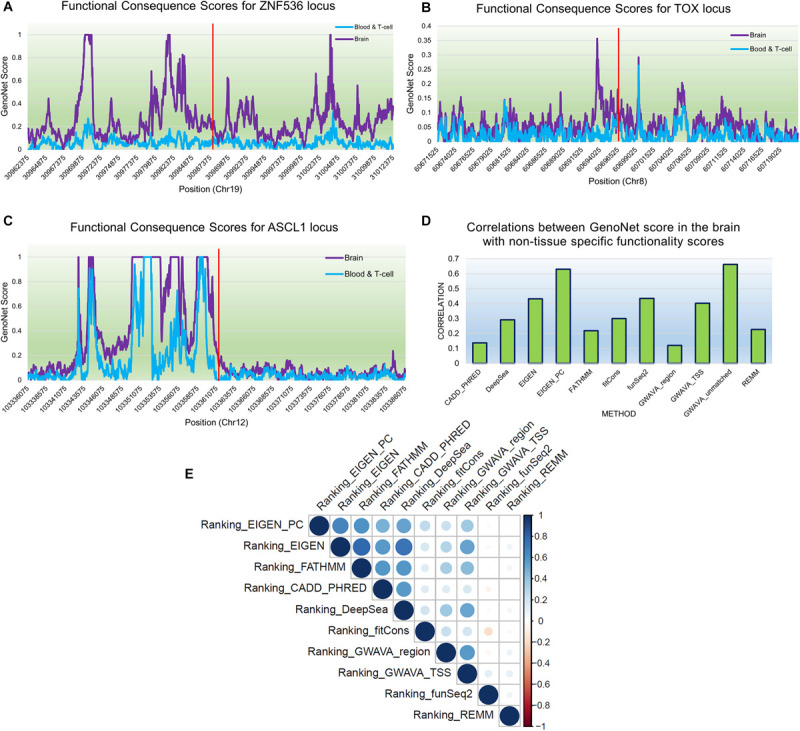
An example of GenoNet scores for three SCZ loci and their flanking regions of 25kb to the SCZ variant in the two tissues of the brain and blood and the correlations between non-context-specific rankings. Red bar represents the position of the SCZ variant. **(A)** Functional consequence scores for ZNF536; **(B)** functional consequence scores for TOX; **(C)** functional consequence scores for ASCL1; **(D)** correlations between 11 non-context-specific functional prediction scores and GenoNet; **(E)** correlations between the obtained rankings from context-free functional predication measures.

We used GenoNet (He et al., 2018) (see section “Materials and Methods”), a semi-supervised approach which jointly utilizes confirmed regulatory variants and millions of unlabeled variants for *in-silico* functional prediction of the SCZ GWAS SNPs in 127 tissues and cell-types from the Roadmap Epigenomics Project (Bernstein et al., 2010). We observed that not all of the SNPs show the highest GenoNet score in brain or neuronal cell-types. We identified 31 SNPs among which 20 showed the highest GenoNet score uniquely in the brain while 11 shared the highest score across several other tissues in addition to the brain (Figure 2). The SNPs localized in intergenic regions showed the highest GenoNet scores in the brain while majority of intronic variants shared the highest scores with the other cell-types or tissues in addition to brain. None of the remaining SCZ SNPs showed higher functional score in the brain. Applying GenoNet to all of the obtained proxy SNPs, we did not observe any proxy SNPs with a higher score in brain cell types than the original SNPs. To present a clearer picture on how cell-specificity can affect functionality of common variants, we selected three loci rs36104021, rs1473594, and rs2053079 where the first two are intergenic SNPs closest to the transcript start site (TSS) of the genes *ASCL1* and *TOX*, respectively, while the third variant is an intronic locus to the gene *ZNF536* (Figures 3A–C). An important observation is that although some of the SCZ variants bear the highest GenoNet score across various cell-types, they do not necessarily gain the highest impact score of 1. For example in Figure 3, the highest GenoNet score for two loci *ZNF536* and *ASCL1* in 50kb window flanking the SCZ loci is 1 while being ∼0.36 in *TOX*. This implies that the functional consequences of SCZ variants may not be the same in every cell type and tissue. We replicated our experiments on the rest of the SCZ loci which did not yield highest GenoNet scores in the brain followed by obtaining all of their GenoNet scores. No proxy SNPs were found to have a higher impact score compared to their queried SNPs.

We additionally used ExPecto (Zhou et al., 2018), a deep learning sequence-based *ab initio* method for predicting effects of variant on the disease risk (see section “Materials and Methods”). In addition tothe SCZ loci, we searched a 100kb window flanking each SNP to account for proxy variants in case the queried SNPs were not available in ExPecto. Out of 145 SNPs, ExPecto returned no results for 90 SNPS (as well as their proxies) while 36 SNPs showed low impact on brain-specific expression levels. Of the top GenoNet hits, *ZNF536* and *CA8* loci were found in ExPecto; however, *BCL11B* and *ZNF823* yielded medium to high impact scores on brain-specific expression scores. *BCL11B* is a zinc finger transcription factor with significant roles in differentiation of neuronal subtypes in the central nervous system (Lennon et al., 2017). *BCL11B* harbors the intronic GWAS SNP rs35604463 associated with SCZ. This gene has also been implicated in patients with neurodevelopmental disorders (Lessel et al., 2018). On the other hand, *ZNF823*, harbors the SCZ GWAS SNP rs72986630 in its 5′-UTR. ZNF536 is a transcription factor which plays an essential role in the development of forebrain neurons and have been implicated in social behaviors (Thyme et al., 2019). In contrast with the other significant genes discussed here, all of loci associated with CA8 are intergenic and closest to this gene. However, CA8 has been implicated to share rare copy number variation (CNV) in unrelated probands with SCZ (Costain et al., 2013).

In conclusion, we did not observe general concordance between context-specific machine learning-based techniques (GenoNet and ExPecto) in this application. Such disparities may arise due toseveral reasons, related to training datasets and underlying methodological differences. As another example, we tested the *MIR137* SCZ risk locus, a well-known risk gene which plays an important role in neuronal development (Mahmoudi and Cairns, 2017) and a well-studied SCZ GWAS locus (Pardinas et al., 2018). GenoNet yielded the highest risk score in the brain while ExPecto does not cover this locus. Although it is not surprising to get inconsistent results across methods, we can use the consistent results from the complementary methods to identify genes that are more likely to be relevant for SCZ.

### Context-Free Fine Mapping of SCZ Susceptibility Loci

A number of context-free methods are available that infer functional importance of genetic variants without considering the cell specificity. Here, we applied 11 widely used predictive scoring metrics on the SCZ loci including: the regulatory Mendelian mutation framework (REMM) (Smedley et al., 2016), genome-wide annotation of variants (GWAVA) (Ritchie et al., 2014) in three different modes, FunSeq2 (Fu et al., 2014), fitCons (Gulko et al., 2015), FATHMM (Shihab et al., 2015), EIGEN and EIGEN_PC (Ionita-Laza et al., 2016), DeepSea (Zhou and Troyanskaya, 2015), and CADD_PHRED (Kircher et al., 2014). A list of the methods used in this study are provided in Table 1, but we acknowledge that these do not cover all available computational methods that were published in the past few years. Notably, we found moderate to weak correlations between the predicted functional impacts of SCZ loci in these context-free methods with the brain-specific GenoNet predicted scores (Figure 3D). EIGEN_PC and GWAVA_unmatched score showed 0.65 Spearman’s rank correlation with the GenoNet scores while the rest of the methods had rank correlations lower than 0.5. As a large portion of the queried loci were not available in ExPecto, we could not calculate the correlations between the context-free measures with ExPecto. With respect to the context-free measures, we found a reasonable agreement among their predictions. Specifically, we found that four measures, including REMM, FunSeq2, GWAVA_region and GWAVA_TSS, deviate from the other functional scores, while the remaining scores showed an average correlation above 0.6.

**TABLE 1:** A list of the methods used in the study.

**Method**	**Mode**	**Platform**
REMM	Context-free	Web
GWAVA (in 3 modes)	Context-free	Web
FunSeq2	Context-free	Web
fitCons	Context-free	Web
FATHMM	Context-free	Web
EIGEN	Context-free	Web
EIGEN_PC	Context-free	Web
DeepSea	Context-free	Web
CADD_PHRED	Context-free	Web
GenoNet	Context-specific	Web
ExPecto	Context-specific	Web

For illustration, we considertwo loci where the first one shows good concordance between context-specific and context-free measures while the second one shows a substantial discordance. For this, we re-iterate SCZ SNP rs2660304, a common variant annotated to *MIR137* SCZ risk gene. This SNP shows the highest GenoNet score in the brain. Notably, this SNP is ranked among the top 10% of all the SCZ queried GWAS SNPs in a majority of context-free measures. On the other hand, rs1353545 annotated to the gene *FHIT* shows a reverse pattern where context-free measures predominantly rank it as a consequential locus while GenoNet does not predict significant functional consequence for this locus in the brain. These observations suggest that care should be taken when applying these methods in diseases that affect very specific tissues (such as SCZ) and we believe that these approaches can play a complementary role in pinpointing functional consequences of common variants.

### Evaluating Consensus Between Computational Methods vs. *in vitro* Models

In an important recent study by Schrode et al. (2019), one putative SCZ GWAS (*FURIN*) hit and four top-ranked SCZ expression quantitative trait loci (eQTLs) for genes *FURIN*, *SNAP91*, *TSNARE1*, and *CLCN3* were studied using CRISPR-mediated gene editing in isogenic human induced pluripotent stem cells. While *FURIN* individually led to significant phenotypic abnormalities in the derived neurons, the other four eQTLs showed significant phenotypic synergy on synaptic functions compared to their individual functional consequences. Our analysis reveals a similar pattern. While the putative causal variant in *FURIN* was ranked among the top 5% of highly functional loci in the brain by GenoNet, it was among the top 5% of the prioritized loci at least in half the context-free measures, while this was not the case for the rest of the loci. For instance, *TSNARE1* was ranked high in the majority of the context-free measures butit ranked quite low in the brain-specific scores from GenoNet. Although their study is among the first combinatorial approaches to study how common variants can serve as *cis*-eQTLs, it provides critical insights intothe additive effects of common variants on pre/post-synaptic functions as well as collective disease-associated gene expression paradigms. With this particular case analysis, we believe that context-specific and context-free measures can act in a complementary fashion. To illustrate this, we showed that the loci with a high tissue-specific functional impact score which also have a high impact score using context-free measures can be considered for further investigation with a higher confidence. Although further experimental tests are required to investigate a larger fraction of the known GWAS loci, our hypothesis is supported by the aforementioned loci tested by Schrode et al. (2019).

## Discussion

Evaluating the functional consequences of common genetic variants by computational methodsis a challenging task, as most of them are non-coding variants. It can be more challenging given that many available computational methods are context-free, i.e., they do not take into account the functional impacts of genetic variants in distinct cell-types or tissues. Novel data-driven approaches have made significant strides to make more accurate predictions based on the tissues being impacted by the disease. To gain insights into the similarities or disparities of these two types of approaches, we have laid the frame work of an analytical pipeline to shortlist high-confidence GWAS loci, or potentially disease-causing SNPs. Evaluating this pipeline on SCZ, as a disease with rich resources of common associated variants, followed by benchmarking the findings with *in-vitro* experiments creates a robust framework for downstream analyses such as CRISPR-mediated experiments.

This framework starts with annotating the lead SNPs and their proxy SNPs and extracting missense, nonsense, intronic, splice-sites, UTRs, and intergenic variants. Then, we applied over two-dozen conventional and context-specific methods on these variants as well as their proxies. As an illustration, we have focused on SCZ where common genetic variants share a large portion of the disease heritability. We took a consensus-based strategy to rank the variants in which we hypothesized that the SNPs with the highest brain-specific scores in context-specific methods which also have a high context-free score are more likely to be disease causal. Overall, we did not find significant overlap between these two sets of approaches for ranking common variants regarding their functional consequences. Yet, we found that variants with high scores in both approaches in the tissue of interest (here brain) manifest strong implications in SCZ.

As the outcome of our analysis, among context-free measures, we ranked each SCZ locus and obtained the average ranking (see “Materials and Methods”) for each locus as well as their significance P-values (Supplementary Table S2). Then, we calculated the Pearson correlation between the obtained rankings for each variant across the entire benchmarked methods (Figure 3E). Two methods funSeq2 and REMM are almost uncorrelated with the rankings of the other methods while fitCons generated less correlated rankings. However, there is strong correlation between the rankings from the remaining context-free functional scores. Focusing on the concordant results from the context-free measures and the state-of-the-art machine learning-based tissue-specific methods, we found several loci including *ZNF823* and *BCL11B* to show the highest functional consequences specifically in the brain given their GenoNet scores. Previously we had found multiple lines of evidence discussed in the previous sections on pathogenicity of these two genes. For example, *ZNF823* and *BCL11B* were the only two loci to be picked up by ExPecto as the most deleterious GWAS hits contributing tothe disease risk in the brain. In a study using genotypes and gene expression levels from CMC, Dobbyn et al. (2018) had re-identified *ZNF823* to be among the GWAS loci to have strong evidence for co-localization with expression quantitative trait loci (eQTL) in brain. In addition to *ZNF823*, four other loci were found in the list provided by Dobbyn et al. (2018) including *FURIN*, *FTCDNL1*, *DCLK3*, and *SNAP91*. These loci showed a GenoNet ranking score in the brain as 1, 1, 40, and 120, respectively (Supplementary Table S2). They showed an average ranking in the context-free methods as 16, 28, 42, and 86, respectively (Supplementary Table S2). Therefore, except for *FTCDNL1* and *FURIN* which show strong consistency between the GenoNet and context-free scores, the other two showed no significant consistency. Signatures of *ZNF823* has also been reported in Down syndrome (Hibaoui et al., 2014) and perturbation of immunological pathways upon vaccination (Stein et al., 2016). On the other hand, *BCL11B* expression has been identified to confirm the T cell-lineage identity of multipotent progenitor cells in the CD4^–^CD8^–^ double-negative pro-T cell DN2 stages (Hosokawa et al., 2018) as well as being identified as an epigenetic regulator of gene expression in SCZ (Whitton et al., 2016). As a result, despite relative consistency between the outcomes of context-free measures, they do not necessarily match the tissue-specific methods. However, shared loci with the highest scores in both context-free and context-specific methods may be the most promising loci.

In conclusion, in this paper we introduced an analytical pipeline to identify disease associated common genetic variants. This pipeline considers disease-associated loci as well as their proxy SNPs to widen the search space to increase the chance of pinpointing the potential causal variants. This pipeline leverages tissue-specific and context-free measures in an ensemble fashion to make an accurate ranking of the potential causal variants. As an illustration, collecting SCZ GWAS loci and flowing them through the pipeline led to a shortlist of loci to generate hypothesis for further functional studies. Using the most recent experimental findings on these loci, we showed that the pipeline can be employed by researchers to prioritize available common variants for downstream analyses.

## Materials and Methods

### List of SCZ Common Variants

We used SCZ GWAS data from the CLOZUK study (Pardinas et al., 2018) which includes 145 variants as well as the PGC study (Schizophrenia Working Group of the Psychiatric Genomics Consortium, 2014). The CLOZUK study is the largest available GWAS of SCZ covering 40,675 cases and 64,643 controls.

### Cell Type-Specific Fine Mapping of SCZ Loci Using GenoNet

We used GenoNet (He et al., 2018), a semi-supervised approach which utilizes logistic elastic-net on thousands of experimentally labeled variants, and incorporates this information with unsupervised predictions on millions of unlabeled variants to improve the prediction accuracy of functional effects. In GenoNet, $\hat{f}$ represents the estimated prediction function:

(1) $$\hat{f}=\underset{f}{\text{argmin}}\sum_{i=1}^{m}l_{p}\,\left(Y_{i},f\,\left({\text{X}}_{i}\right)\right)+\gamma_{I}\,\sum_{i=1}^{l}{\left({\hat{Y}}_{i}^{u}-f\,\left({\text{X}}_{i}\right)\right)}^{2}$$

in which *l_p* denotes the penalized log-likelihood for the labeled data, *Y*_*i*_ ∈ {0,1} are the labels for *m* variants with validated labels, ${\hat{Y}}_{i}^{u}\in \left[0,1\right]$ represent the predicted values for a large number (*l*) of mutations from a prior unsupervised method, **X**_*i*_ denotes the functional annotations, γ_*I*_ is a tuning parameter which is used to maximize the area under receiver operating characteristics curve (AUROC). FUN-LDA score (Backenroth et al., 2018) is used in GenoNet given that it is one of the most reliable tissue specific genome-wide functional scores. GenoNet adopts Elastic-net as its supervised algorithm given its superior performance when the features are correlated with sparse non-zero coefficients (He et al., 2018).

### ExPecto for *ab initio* Prediction of Variant Effects on Expression and Disease Risk

We utilized ExPecto web portal^1^ to predict the effects of the SCZ SNPs across tissues and cell-types. In addition to the SCZ loci, we queried a 100kb flanking each SNP. 52 tissues from Epigenome Roadmap Data in ExPecto were used for fine mapping. ExPectois a sequence-based expression prediction framework with three sequentially acting components including: (a) a deep neural network to model epigenomic effects being trained by the sequencing data to predict the probabilities for some epigenetic markers such as histone marks at each position etc.; (b) a series of spatial transformation functions to summarize the predicted pattern within the chromatin profiles aimed at finding a reduced set of features; (c) using the set of spatially transformed features to predict tissue-specific gene expression predictions using regularized linear models.

### Annotation of the Common Variants and Their Proxy Loci

We used LDproxy (Machiela and Chanock, 2015) to find proxy SNPs to the SCZ GWAS loci. Correlation threshold of *R*^2^ > 0.5 is used for specifying the proxy SNPs. LDproxy utilizes reference haplotypes from 26 different population groups from Phase 3 of the 1000 Genomes Project (Genomes Project et al., 2012). European populationhas been used throughout the study. We used ANNOVAR (Wang et al., 2010), to annotate the SCZ variants to the human reference genome hg19.

### Context-Free Functional Prediction Methods

Functional prediction measures of multiple different methods were calculated using SNPnexus IW-Scoring (Wang et al., 2018). SNPnexus is collection of multiple context-free methods that we used to run most of the context-free methods in this study. Batch query option using GRCh37/hg19 reference genome was employed on all of the SCZ SNPs and their proxies on all of the Non-coding Scoring schemes. The regulatory Mendelian Mutation (REMM) framework (Smedley et al., 2016) is a machine learning-based method to predict the causality of arbitrary positions in the non-coding regions of the human genome in developing Mendelian diseases if mutated. DeepSEA (Zhou and Troyanskaya, 2015) is a deep learning algorithmic framework to predict the chromatin effects of sequence alterations with single nucleotide sensitivity. DeepSEA is designed to predict the epigenetic states of a sequence and utilize this capability to prioritize genetic variants. GWAVA (Ritchie et al., 2014) is a prediction model which uses a modified version of the random forest algorithm to prioritize non-coding variants by integrating various genomic and epigenomic annotations. Eigen and Eigen_PC (Ionita-Laza et al., 2016) are unsupervised approaches to integrate different annotations intoone measure of functional score without using any labeled data. Eigen generates a meta-score using benign and putatively disease-associated variant from published studies. FATHMM (Shihab et al., 2015) is a framework for functional prediction of coding and non-coding sequence variants. FATHMM uses various published genomic annotations followed by using kernel integration to weight the significance of each annotation score. FunSeq2 (Fu et al., 2014), a computational framework to annotate and prioritize non-coding variants, integrates genomic resources with a streamlined variant-prioritization pipeline which contains a weighted scoring scheme to combine loss/gain of function events, network centrality, inter/intra-species conservation, and per-element recurrence across samples. fitCons “fitness consequence” (Gulko et al., 2015) is a scoring method to estimate the probability of a point mutation at each position in the genome to influence fitness. CADD_PHRED (Kircher et al., 2014) is a method for objective integration of diverse annotations into a single score for each variant. This method has been based on training a support vector machine to differentiate millions of human-derived high-frequency alleles from millions of simulated variants.

### Average Ranking of SCZ GWAS Loci in Context-Free Measures

For each of the context-free functional prediction methods (*m*=11), we obtained the prediction scores and ranked the loci based on the outcome of each method. Next, we averaged the ranking of the methods to create an ultimate ranking measure for each loci as follows:

(2) $$A\,v\,e\,r\,a\,g\,e\,R\,a\,n\,k\,i\,n\,g_{i}=\frac{\sum_{j=1}^{m}R_{i\,j}}{m}$$

where *R*_*ij*_ denotes the ranking of the SNP *i* by the method *j*, *i*={1,…,145} denotes the GWAS loci *i* and *m*={1,…,11} represents the functional prediction method *m*. Note that we only take the average ranking of context-free methods for each locus. Upon obtaining the average ranking for each loci, we made a final ranking and compared them directly with the rankings of the same loci from tissue-specific measures.

## Data Availability Statement

Publicly available datasets were analyzed in this study. This data can be found here: CLOZUK Consortium and PGC Consortium.

## Author Contributions

ADT conceived the study, designed the pipeline, conducted the experiment, and wrote the manuscript. II-L conceived the study, analyzed the results, and edited the manuscript. KW supervised the study, provided technical support, and edited the manuscript. All authors contributed to the article and approved the submitted version.

## Conflict of Interest

The authors declare that the research was conducted in the absence of any commercial or financial relationships that could be construed as a potential conflict of interest.

## Footnotes

1 https://hb.flatironinstitute.org/expecto/
